# Complete Response Case of Metastatic Hormone-Sensitive Prostate Cancer Treated With Triplet Therapy (Androgen Deprivation Therapy + Docetaxel + Darolutamide)

**DOI:** 10.7759/cureus.81642

**Published:** 2025-04-03

**Authors:** Tatsuya Shimomura, Fumihiko Urabe, Hajime Onuma, Keigo Sakanaka, Soshi Kadena, Yuma Goto, Katsuki Muramoto, Mana Nakata, Nozomu Furuta, Takahiro Kimura

**Affiliations:** 1 Urology, Jikei University School of Medicine, Tokyo, JPN

**Keywords:** adt (androgen deprivation therapy), darolutamide, docetaxel, mhspc, radiological complete response, triplet therapy

## Abstract

In this work, we report a complete response case of a 69-year-old male with metastatic prostate cancer (initial prostate-specific antigen (PSA)=26.3 ng/mL, Gleason score 3+4=7, cT3a, N0, M1b) treated with triplet therapy comprising leuprorelin + docetaxel (75 mg/m^2^/3 weeks) + darolutamide (1,200 mg/d). His PSA was checked monthly, and radiological scans (computed tomography and bone scans) were administered every three months. His PSA declined quickly, reaching <0.01 ng/mL five months after starting triplet therapy, but his bone scan index declined gradually, reaching 0.0% 13 months after starting triplet therapy. To evaluate the true efficacy of triplet therapy, regular radiological testing should be performed, similar to regular PSA testing.

## Introduction

Prostate cancer is the most common cancer in men, both in the US and worldwide [1,2], with the first line of treatment usually being androgen deprivation therapy (ADT). However, cancer cells can become resistant to ADT after one to four years, resulting in a progressive form of prostate cancer called castration-resistant prostate cancer (CRPC), which is characterized by poor long-term survival rates [3]. To prevent CRPC status and prolong survival outcome, six major RCTs with upfront docetaxel and androgen-receptor signaling inhibitors (ARSIs) have shown good survival benefits in treatment arms against metastatic hormone-sensitive prostate cancer (mHSPC) [4-9].

Recent National Comprehensive Cancer Network guidelines recommend ARSI upfront doublet treatment as a first-line treatment for mHSPC. In the ARASENS trial, upfront triplet therapy (ADT + docetaxel + darolutamide) exhibited better survival results against mHSPC than doublet therapy (ADT + docetaxel) [10], leading to Food and Drug Administration approval of triplet therapy for mHSPC. Although triplet therapy is approved worldwide, its treatment efficacy in practice is still being investigated. Herein, we report a complete response case of metastatic prostate cancer treated with triplet therapy.

## Case presentation

**Figure 1:**
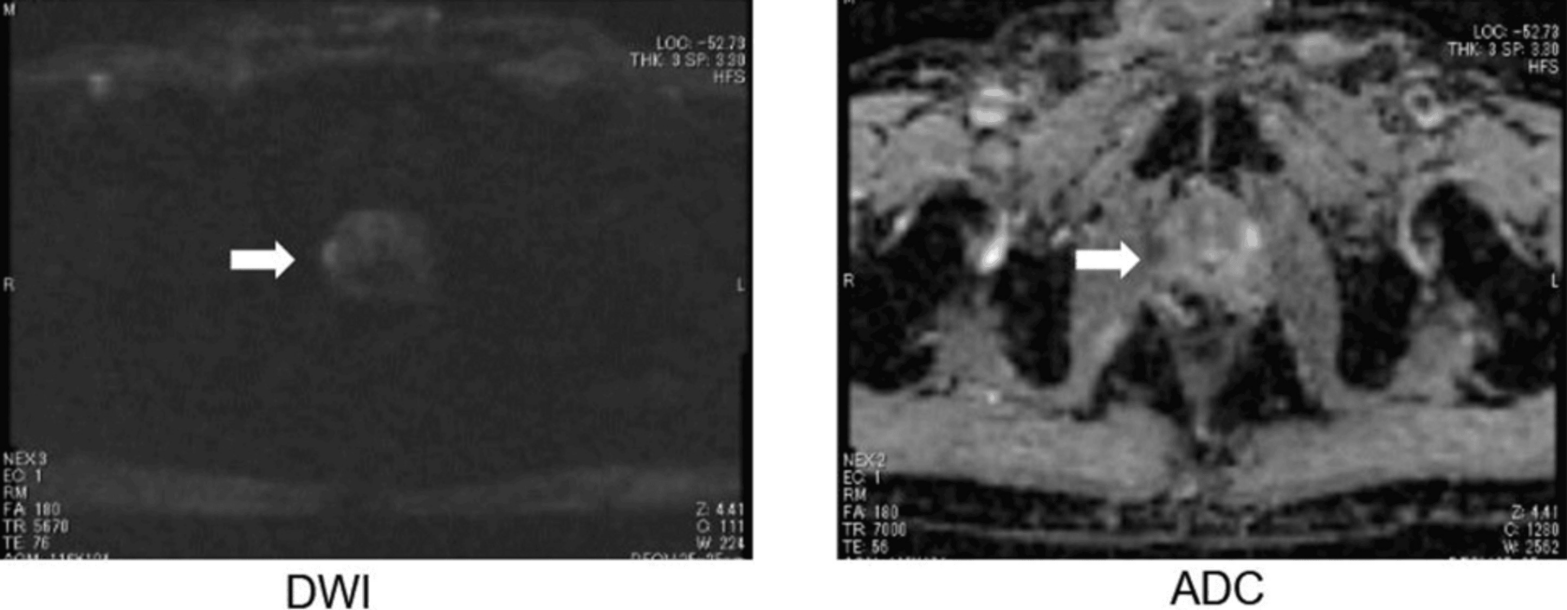
MRI imaging of the prostate at initial diagnosis Left: diffusion-weighted imaging (DWI); Right: apparent diffusion coefficient (ADC) map

A 69-year-old male, with suspected prostate cancer, presented with an initial prostate-specific antigen (PSA) level of 26.3 ng/mL. A prostate biopsy revealed prostate adenocarcinoma with a Gleason score of 3+4=7 (i.e., four cores positive out of 12 cores). Prostate magnetic resonance imaging (MRI) showed prostate carcinoma with extraprostatic extension (Figure 1).

**Figure 2:**
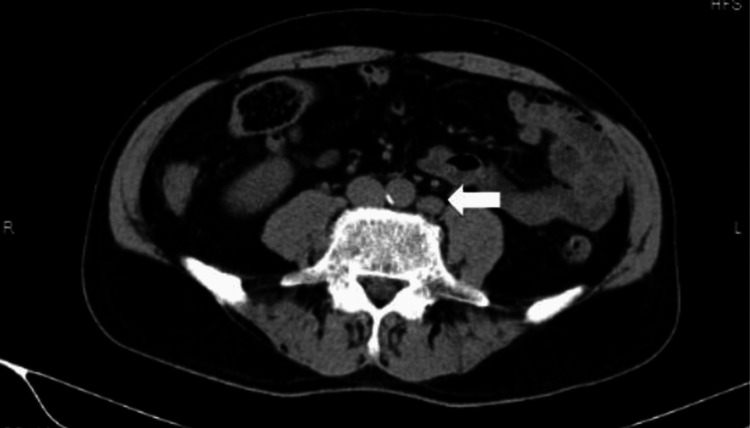
CT scan imaging of the abdomen at initial diagnosis Arrow head: swelling paraaortic lymph node

A CT scan showed swelling of a paraaortic lymph node, 2 cm in diameter (Figure 2).

**Figure 3:**
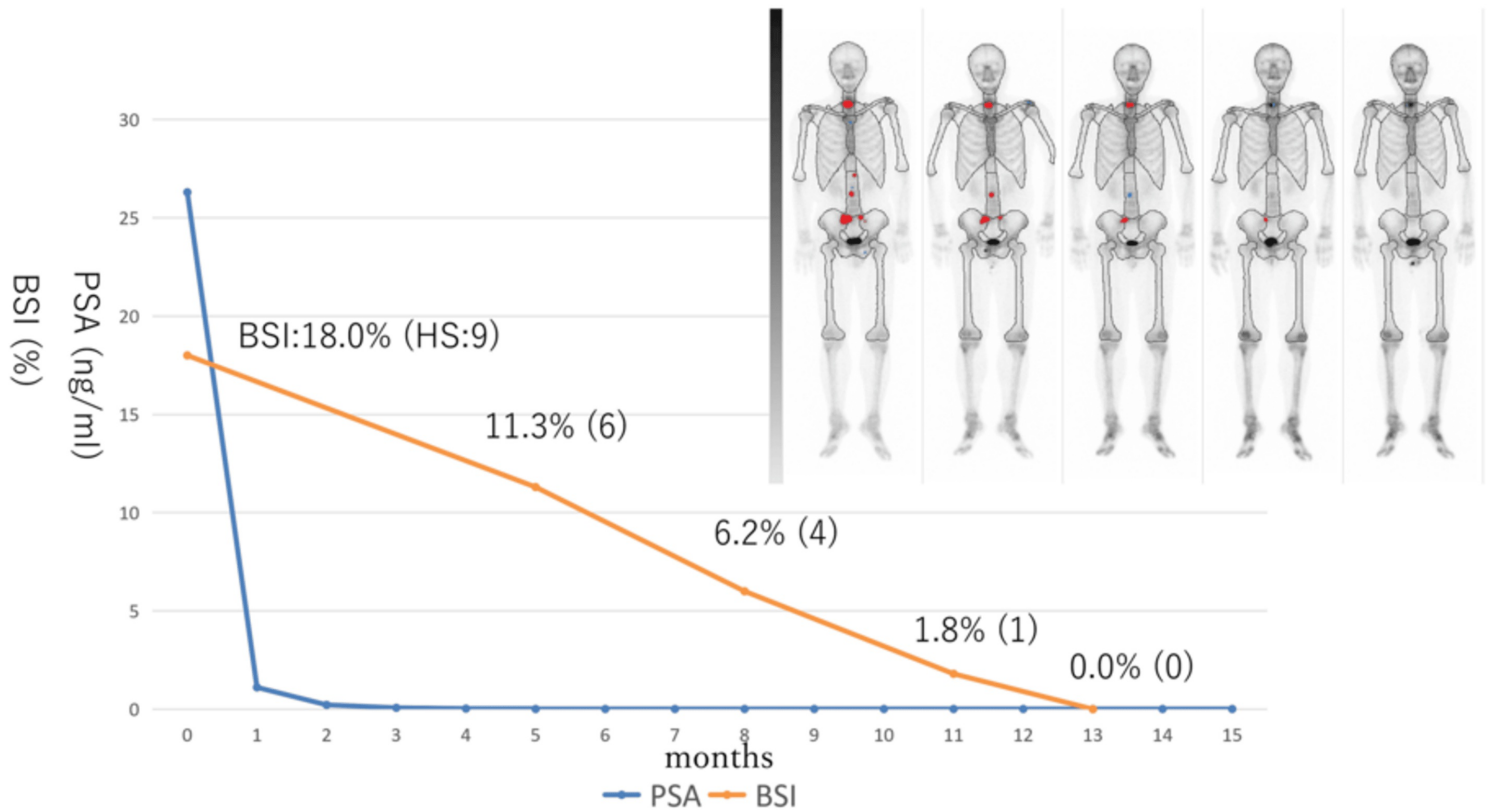
Changes of PSA and BSI and bone scan imaging after starting triplet therapy BSI: bone scan index, HS: number of hotspots

A bone scan showed multiple bone metastases, with nine hot spots and a bone scan index (BSI) of 18.0% (Figure 3, leftmost).

The patient was diagnosed with prostate cancer (clinical stage: cT3a, N0, M1ab). The patient was started on ADT (leuprorelin) + docetaxel (75 mg/m^2^) + darolutamide (1,200 mg/d). His PSA was checked monthly, and radiological checks (CT scan and bone scan) were performed every three months. Because febrile neutropenia occurred at the first course of docetaxel, docetaxel was administered every four weeks, and doses were reduced (80 mg/body). After docetaxel treatment was completed, ADT + darolutamide were continued. No other adverse events occurred. Changes in PSA and BSI during the clinical course and imaging of bone scans are shown in Figure 3.

**Figure 4:**
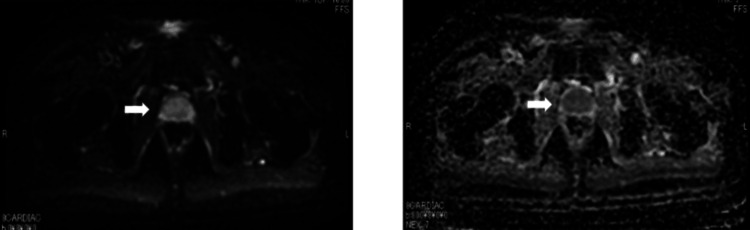
MRI imaging of the prostate at 14 months after starting triplet therapy Left: diffusion-weighted imaging (DWI); Right: apparent diffusion coefficient (ADC) map

**Figure 5:**
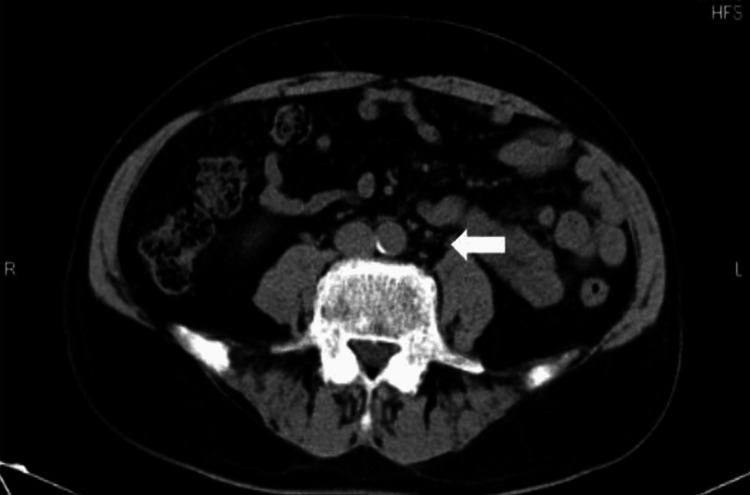
CT scan imaging of the abdomen at 13 months after starting triplet therapy Arrow head: shrinking paraaortic lymph node

PSA declined quickly and reached <0.01 ng/mL in five months after starting triplet therapy. In terms of the BSI, it declined gradually and reached 0.0% in 13 months after starting triplet therapy. At this time, MRI (Figure 4) revealed no evidence of prostate cancer, and CT (Figure 5) revealed shrunken lymph nodes, which were judged to have no evidence of metastasis by the radiologist. His PSA declined quickly and reached <0.01 ng/mL five months after starting triplet therapy. The BSI declined gradually, reaching 0.0% 13 months after starting triplet therapy.

Follow-up time was 15 months from starting triplet therapy, and ADT + darolutamide were continued.

## Discussion

The ARASENS study showed that triplet therapy (ADT + docetaxel + darolutamide) had better survival benefits than doublet therapy (ADT + docetaxel) [10], where PSA response, progression-free survival, and overall survival were evaluated as endpoints. However, radiographic responses were unknown. Recently, our group reported real-world clinical outcomes of triplet therapy (ADT + docetaxel + darolutamide) against mHSPC in terms of treatment efficacy, with good PSA and radiographic responses [11]. Additionally, 95.6% of patients saw their PSA decline by >90%, and 97.8% of cases were radiologically stable with good response. We followed triplet cases and experienced radiographic CR cases, so we reported this case here. To the best of our knowledge, this is the first report of a complete response case with triplet therapy. Of clinical importance, in this case, although PSA decreased quickly and reached <0.01 ng/mL after five months, the decrease in BSI occurred more slowly, reaching 0.0% after 13 months, which can be explained by a difference in kinetics between PSA and BSI. PSA response is usually observed to evaluate the clinical efficacy of the treatment agent, with PSA kinetics investigated after initiating the agent. However, radiographic changes are often under-evaluated, and radiographic response rates are not well known in real-world clinical practice. BSI can also be used to objectively evaluate, and its changes are able to be quantified like PSA. In this case, as we performed radiographic testing (CT scan and bone scan) every three months after starting triplet therapy, we were able to evaluate the kinetics of both PSA and BSI. Future studies should further investigate the relationship between PSA response and BSI response to determine the true efficacy of triplet therapy and the biological features of metastatic prostate cancer, as radiographic progression may occur without PSA progression more than is expected [12].

## Conclusions

Herein, we reported a radiographic complete response case of a patient with mHSPC treated via triplet therapy. Triplet therapy was considered effective for mHSPC. Regular radiological testing is recommended, similar to regular PSA testing, to evaluate the true efficacy of triplet therapy.
